# Supplementary material for: Modeling glioblastoma heterogeneity as a dynamic network of cell states
Source: Mol Syst Biol. 2021 Sep 16;17(9):e10105. doi: 10.15252/msb.202010105 (PMC8444284; doi:10.15252/msb.202010105)
Supplement: Supplementary file 6 — Source Data for Figure 5 [file MSB-17-e10105-s004.zip › Figure5A_sourcedata/GSEA_3071/hallmarks_stateI.GseaPreranked.1623742531165/HALLMARK_P53_PATHWAY.html]

Details for gene set HALLMARK\_P53\_PATHWAY[GSEA]

|  || Dataset | state13071 |
| Phenotype | NoPhenotypeAvailable |
| Upregulated in class | na\_pos |
| GeneSet | HALLMARK\_P53\_PATHWAY |
| Enrichment Score (ES) | 0.356677 |
| Normalized Enrichment Score (NES) | 1.984614 |
| Nominal p-value | 0.0 |
| FDR q-value | 0.0042552147 |
| FWER p-Value | 0.024 |
Table: GSEA Results Summary

  

Fig 1: Enrichment plot: HALLMARK\_P53\_PATHWAY      
 Profile of the Running ES Score & Positions of GeneSet Members on the Rank Ordered List

  

| PROBE | GENE SYMBOL | GENE\_TITLE | RANK IN GENE LIST | RANK METRIC SCORE | RUNNING ES | CORE ENRICHMENT || 1 | KRT17 |  |  | 20 | 1.593 | 0.0335 | Yes |
| 2 | NUPR1 |  |  | 21 | 1.585 | 0.0726 | Yes |
| 3 | SLC7A11 |  |  | 26 | 1.530 | 0.1092 | Yes |
| 4 | S100A4 |  |  | 30 | 1.506 | 0.1455 | Yes |
| 5 | PHLDA3 |  |  | 93 | 1.116 | 0.1551 | Yes |
| 6 | ZFP36L1 |  |  | 97 | 1.096 | 0.1812 | Yes |
| 7 | F2R |  |  | 105 | 1.067 | 0.2055 | Yes |
| 8 | CDKN2B |  |  | 112 | 1.047 | 0.2296 | Yes |
| 9 | PDGFA |  |  | 119 | 1.015 | 0.2529 | Yes |
| 10 | TRIB3 |  |  | 159 | 0.894 | 0.2637 | Yes |
| 11 | TGFB1 |  |  | 165 | 0.884 | 0.2841 | Yes |
| 12 | KIF13B |  |  | 182 | 0.855 | 0.3005 | Yes |
| 13 | TM4SF1 |  |  | 249 | 0.735 | 0.2995 | Yes |
| 14 | JAG2 |  |  | 255 | 0.730 | 0.3161 | Yes |
| 15 | SOCS1 |  |  | 292 | 0.686 | 0.3226 | Yes |
| 16 | SLC3A2 |  |  | 324 | 0.651 | 0.3296 | Yes |
| 17 | PROCR |  |  | 355 | 0.627 | 0.3364 | Yes |
| 18 | EPHX1 |  |  | 429 | 0.570 | 0.3293 | Yes |
| 19 | ABHD4 |  |  | 462 | 0.539 | 0.3333 | Yes |
| 20 | OSGIN1 |  |  | 470 | 0.535 | 0.3445 | Yes |
| 21 | CTSD |  |  | 474 | 0.531 | 0.3567 | Yes |
| 22 | HMOX1 |  |  | 623 | 0.443 | 0.3246 | No |
| 23 | S100A10 |  |  | 673 | 0.422 | 0.3208 | No |
| 24 | VDR |  |  | 681 | 0.420 | 0.3292 | No |
| 25 | KLF4 |  |  | 742 | 0.400 | 0.3216 | No |
| 26 | DDIT3 |  |  | 749 | 0.398 | 0.3297 | No |
| 27 | FOS |  |  | 832 | 0.370 | 0.3151 | No |
| 28 | DDIT4 |  |  | 865 | 0.361 | 0.3147 | No |
| 29 | CASP1 |  |  | 981 | 0.331 | 0.2895 | No |
| 30 | CDKN2A |  |  | 983 | 0.331 | 0.2973 | No |
| 31 | CD81 |  |  | 1059 | 0.315 | 0.2834 | No |
| 32 | ITGB4 |  |  | 1125 | 0.302 | 0.2719 | No |
| 33 | MXD1 |  |  | 1151 | 0.298 | 0.2720 | No |
| 34 | MXD4 |  |  | 1217 | 0.285 | 0.2602 | No |
| 35 | ISCU |  |  | 1266 | 0.274 | 0.2530 | No |
| 36 | BLCAP |  |  | 1387 | 0.254 | 0.2245 | No |
| 37 | CCND3 |  |  | 1482 | -0.255 | 0.2035 | No |
| 38 | ZMAT3 |  |  | 1575 | -0.265 | 0.1833 | No |
| 39 | RB1 |  |  | 1771 | -0.290 | 0.1339 | No |
| 40 | RPL36 |  |  | 1872 | -0.301 | 0.1123 | No |
| 41 | NOTCH1 |  |  | 1878 | -0.301 | 0.1183 | No |
| 42 | CDK5R1 |  |  | 1879 | -0.302 | 0.1257 | No |
| 43 | MDM2 |  |  | 1904 | -0.305 | 0.1263 | No |
| 44 | CDKN1A |  |  | 2083 | -0.332 | 0.0828 | No |
| 45 | HSPA4L |  |  | 2105 | -0.335 | 0.0850 | No |
| 46 | CDKN2AIP |  |  | 2115 | -0.337 | 0.0907 | No |
| 47 | DCXR |  |  | 2157 | -0.344 | 0.0873 | No |
| 48 | HRAS |  |  | 2276 | -0.367 | 0.0621 | No |
| 49 | BTG2 |  |  | 2318 | -0.375 | 0.0594 | No |
| 50 | TRAF4 |  |  | 2321 | -0.376 | 0.0681 | No |
| 51 | WWP1 |  |  | 2330 | -0.379 | 0.0752 | No |
| 52 | RALGDS |  |  | 2380 | -0.389 | 0.0705 | No |
| 53 | ABAT |  |  | 2383 | -0.390 | 0.0796 | No |
| 54 | BMP2 |  |  | 2589 | -0.436 | 0.0308 | No |
| 55 | LDHB |  |  | 2597 | -0.438 | 0.0396 | No |
| 56 | BAX |  |  | 2660 | -0.457 | 0.0329 | No |
| 57 | FOXO3 |  |  | 2696 | -0.471 | 0.0344 | No |
| 58 | PLK2 |  |  | 2720 | -0.476 | 0.0395 | No |
| 59 | APP |  |  | 2770 | -0.495 | 0.0375 | No |
| 60 | CYFIP2 |  |  | 3063 | -0.645 | -0.0314 | No |
| 61 | FBXW7 |  |  | 3082 | -0.662 | -0.0203 | No |
| 62 | RGS16 |  |  | 3134 | -0.708 | -0.0176 | No |
| 63 | CD82 |  |  | 3261 | -0.870 | -0.0327 | No |
| 64 | CCP110 |  |  | 3262 | -0.870 | -0.0112 | No |
| 65 | PCNA |  |  | 3319 | -0.963 | -0.0037 | No |
| 66 | NINJ1 |  |  | 3372 | -1.108 | 0.0085 | No |
| 67 | CDH13 |  |  | 3414 | -1.277 | 0.0282 | No |
Table: GSEA details [plain text format]

  

Fig 2: HALLMARK\_P53\_PATHWAY: Random ES distribution      
 Gene set null distribution of ES for **HALLMARK\_P53\_PATHWAY**
